# Peaks and troughs: variations in the availability of therapeutic drug monitoring in critical care units across London

**DOI:** 10.1186/2197-425X-3-S1-A397

**Published:** 2015-10-01

**Authors:** KE Grailey, JM Singleton, JB Simon, JLC Wong

**Affiliations:** Barts Health NHS Trust, London, United Kingdom; Imperial College London, London, United Kingdom

## Introduction

Therapeutic Drug Monitoring (TDM) is an evolving tool used to optimise the administration of antimicrobial agents; which are well documented to have toxic side effects [[1]]. In combination with increasing antimicrobial resistance and emphasis on cost-efficiency, provision of the optimal dose of antimicrobial therapy is a very attractive prospect.

Pharmacokinetic (PK) indices can be used to classify the efficacy of an antimicrobial agent. Critical illness may alter a patients PK behaviour [[2]] and therefore these patients may require alternative dosing regimens for optimal management [[3]].

## Objectives

The aim was to evaluate the current use of TDM in Intensive Care Units (ICU's) across London, focusing on the availability of results, the range of drugs for which TDM is offered and presence of critical care specific regimens.

## Methods

A 6 part questionnaire was developed, and data collection was achieved by telephone interviews with Critical Care Pharmacists at 27 London Hospitals between February and April 2015.

## Results

All hospitals offered TDM, however there were significant differences in the application of this tool. TDM was reported to be available for Gentamicin, Vancomycin and Amikacin at almost all sites (27 units [100%]; 26 units [96%] and 19 units [70%] respectively), with the majority providing onsite TDM.

Gentamicin was universally available within 6 hours (27 units [100%]). Vancomycin and Amikacin were frequently available within 6 hours (20 units [74%] and 12 units [44%] respectively). Teicoplanin was available within 6 hours in 5 units [20%] yet took up to 24 hours in 4 units [15%].

For commonly used drugs (Gentamicin, Vancomycin and Amikacin) most units followed Trust Specific Guidelines (27 units [100%], 26 units [96%] and 25 units [93%] respectively), but ICU specific guidelines were only available in 6 units [23%] for Gentamicin and 7 units [27%] for Amikacin. Vancomycin had ICU specific guidelines in 19 units [73%].

## Conclusions

This initial survey across London demonstrates that whilst commonly used antimicrobial agents are often used with TDM to guide therapy, there is still large variation in the range of antimicrobials for which TDM is available. There was also shown to be discrepancy in the availability of test results, which may impact on patient care if potentially toxic or sub-therapeutic levels take 24 hours to be detected. It was also interesting to note that critical care specific guidelines were rare, despite the potential for pharmacokinetic differences in this patient group.

**Figure 1:**
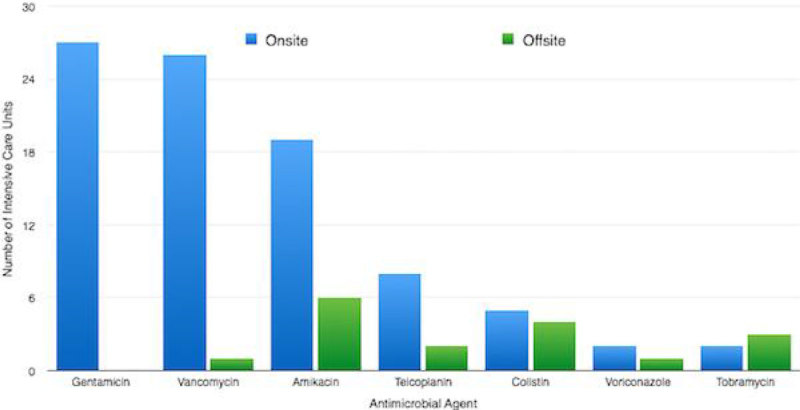
**Number of Intensive Care Units offering TDM for each Antimicrobial Agent, separated according to onsite availability.**

**Figure 2:**
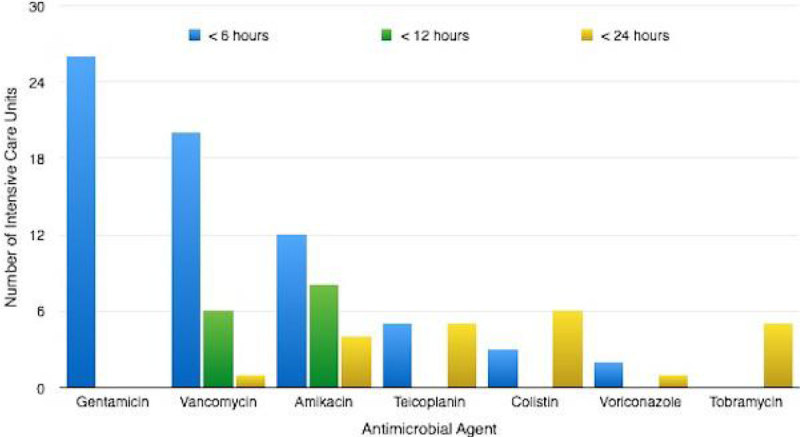
**Availability of therapeutic Drug Monitoring- time for results to be accessible according to antimicrobial agent.**

**Figure 3:**
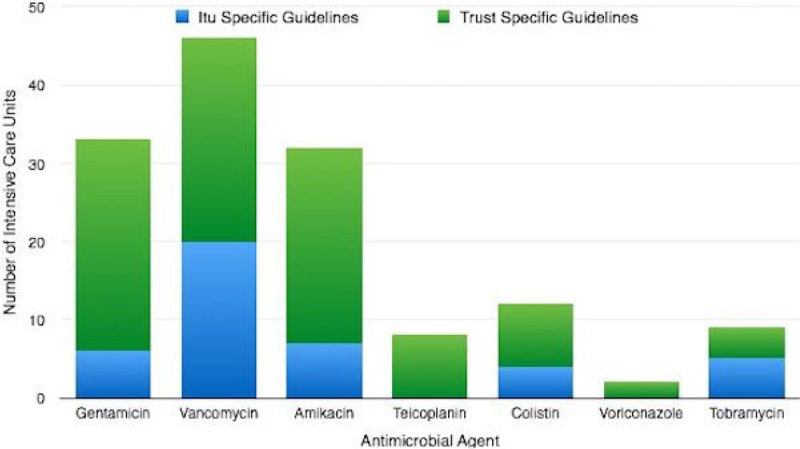
**Availability of Guidelines for therapeutic Drug Monitoring. Chart demonstrates ITU and Trust specific guidelines according to antimicrobial agent.**
